# Dynamic regulation and key roles of ribonucleic acid methylation

**DOI:** 10.3389/fncel.2022.1058083

**Published:** 2022-12-19

**Authors:** Jia Zou, Hui Liu, Wei Tan, Yi-qi Chen, Jing Dong, Shu-yuan Bai, Zhao-xia Wu, Yan Zeng

**Affiliations:** ^1^Community Health Service Center, Geriatric Hospital Affiliated to Wuhan University of Science and Technology, Wuhan, China; ^2^Brain Science and Advanced Technology Institute, School of Medicine, Wuhan University of Science and Technology, Wuhan, China; ^3^School of Public Health, Wuhan University of Science and Technology, Wuhan, China; ^4^Community Health Service Center, Wuchang Hospital, Wuhan, China

**Keywords:** RNA methylation, *N*^6^-methyladenosine, 5-methylcytosine, 5-hydroxymethylcytosine, *N*^1^-methyladenosine, pseudouridine

## Abstract

Ribonucleic acid (RNA) methylation is the most abundant modification in biological systems, accounting for 60% of all RNA modifications, and affects multiple aspects of RNA (including mRNAs, tRNAs, rRNAs, microRNAs, and long non-coding RNAs). Dysregulation of RNA methylation causes many developmental diseases through various mechanisms mediated by *N*^6^-methyladenosine (m^6^A), 5-methylcytosine (m^5^C), *N*^1^-methyladenosine (m^1^A), 5-hydroxymethylcytosine (hm^5^C), and pseudouridine (Ψ). The emerging tools of RNA methylation can be used as diagnostic, preventive, and therapeutic markers. Here, we review the accumulated discoveries to date regarding the biological function and dynamic regulation of RNA methylation/modification, as well as the most popularly used techniques applied for profiling RNA epitranscriptome, to provide new ideas for growth and development.

## 1 Introduction

**FIGURE 1:**
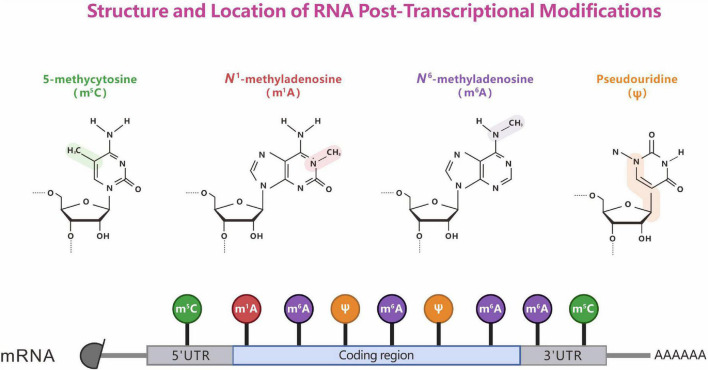
Structure and location of representative RNA post-transcriptional modifications. The chemical properties of RNA modifications **(top)**. The known sites **(bottom)** of m^6^A, m^1^A, m^5^C, and Ψ modification on mRNA.

Ribonucleic acid (RNA) modification plays an important role in linking deoxyribonucleic acid (DNA) to proteins during the transmission of genetic information. More than 100 post-transcriptional RNA modifications have been identified in important biological processes in viruses, archaea, bacteria, and eukaryotes. Methylation accounts for 60% of the total RNA modifications. RNA methylation was discovered as early as the 1970s (Cantara et al., 2011), and it is widely distributed in messenger RNA (mRNA), transfer RNA (tRNA), ribosomal RNA (rRNA), small nuclear RNA (snRNA), small nucleolar RNA (snoRNA), and micro RNA (El Yacoubi et al., 2012). Currently, various distinct modifications in natural RNA have been characterized: *N*^1^-methyladenosine (m^1^A), *N*^6^-methyladenosine (m^6^A), *N*^6^, 2′-*O*-dimethyladenosine (m^6^Am), 5-methylcytosine (m^5^C), 5-hydroxymethylcytosine (hm^5^C), pseudouridine (Ψ), and Adenosine to inosine editing (A-to-I editing), etc. (Roundtree et al., 2017a; Walkley and Li, 2017; Zhao et al., 2020). Among these, m^6^A, m^5^C, m^1^A, and Ψ have been well studied (Figure 1). Compared with DNA methylation, RNA methylation is more complex and diverse, participates in the regulation of many more biological processes, and determines the diversification of RNA post-transcriptional modifications. Mutations in approximately half of the currently known RNA methylation enzymes have been linked to human diseases, including cancer, cardiovascular diseases, congenital genetic disabilities, metabolic diseases, neurological disorders, and mitochondrial-related defects (Jonkhout et al., 2017; Barbieri and Kouzarides, 2020). Here, we review the discoveries to date regarding the dynamic regulation and key roles of RNA methylation.

## 2 Biological patterns of RNA methylation/modification

### 2.1 *N*^6^-methyladenosine (m^6^A)

**FIGURE 2:**
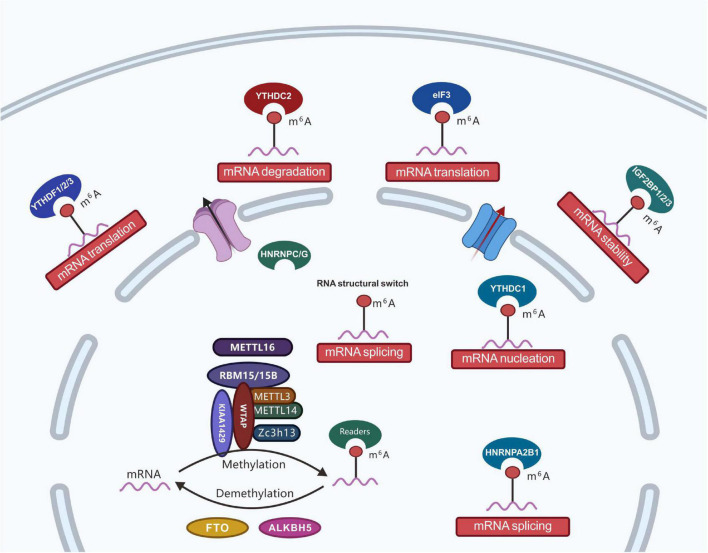
Regulatory roles of m^6^A effector proteins. The m^6^A effectors, including writer proteins (i.e., m6A methyltransferase complex: core subunits METTL3 and METTL14 and additional adaptors proteins including WTAP, ZC3H13, VIRMA, METTL16, RBM15/15B, HAKAI, and KIAA1429); eraser proteins (i.e., RNA demethylases: FTO and ALKBH5), and reader (three classes of reader proteins: ➀ YTH-domain-containing proteins, including YTHDC1, YTHDC2, YTHDF1, YTHDF2, and YTHDF3; ➁ proteins favoring RNA-binding events, including HNRNPA2B1, HNRNPC, and HNRNPG; ➂ RNA binding proteins, including eIF3, IGF2BP1, IGF2BP2, and IGF2BP3). With the development of RNA modification detection technology, m^6^A modifications have been determined to functionally regulate the transcriptome of eukaryotes and processes, such as mRNA stability, splicing, nucleation, localization, and translation.

*N*^6^-methyladenosine (m^6^A), the most abundant methylation modification in eukaryotic mRNA, is the most thoroughly studied type of RNA modification (Schwartz et al., 2013). m^6^A has been found in many eukaryotes, ranging from yeast, Arabidopsis, and *Drosophila* to mammals and even in viruses (Krug et al., 1976; Schwartz et al., 2013; Luo et al., 2014; Lin et al., 2017; Guo et al., 2018). In mammals, m^6^A is widely distributed in multiple tissues, with the highest level of m^6^A in the brain, kidney, and liver (Meyer et al., 2012). Although the existence of m^6^A on RNA was identified as early as the 1970s, little was known about its precise location, temporal dynamics, and regulation until 2011 (Desrosiers et al., 1974). With the identification of the first demethylase fat mass and obesity-associated protein (FTO) of RNA m^6^A and the development of antibody enrichment and high-throughput sequencing technology, accurate mapping of the transcriptome-level m^6^A distribution has been achieved (Jia et al., 2011). The sequence near the m^6^A methylation site on mRNA is highly conserved and mainly occurs on the adenine of RRm^6^ACH, where R = G/A (G > A) and H = U/A/C (U > A > C) (Harper et al., 1990). In addition, whole-transcriptome m^6^A sequencing and comprehensive bioinformatics analysis of human and mouse samples revealed that m^6^A modifications are species-specific (Liu et al., 2020a). m^6^A methylation is a reversible dynamic modification in many species and is jointly regulated by methyltransferases (writers), demethylases (erasers), and binding proteins (readers). The main components of the methyltransferase complex that have been identified include methyltransferase 3 (METTL3), methyltransferase 14 (METTL14), Wilms’ tumor 1-associating protein (WTAP), and KIAA1429 (vir-like m^6^A methyltransferase associated) (Oerum et al., 2021). The demethylases FTO and AlkB homolog 5 (ALKBH5) can reverse methylation (Figure 2; Liu et al., 2013; Zheng et al., 2013).

The biological function of m^6^A is mainly through the post-transcriptional regulation of RNA by m^6^A-binding proteins. Currently known binding proteins include the YT521-B homology (YTH) domain proteins (YTHDF1, YTHDF2, YTHDF3, YTHDC1, and YTHDC2) and nuclear heterogeneous ribonucleoprotein HNRNP family proteins (HNRNPA2B1, HNRNPC, and HNRNPG) (Alarcón et al., 2015; Roundtree et al., 2017b). With the development of RNA modification detection technology, m^6^A modifications have been determined to functionally regulate the transcriptome of eukaryotes and processes such as mRNA stability, splicing, nucleation, localization, and translation (Figure 2). Furthermore, m^6^A is involved in a variety of biological processes such as stem cell differentiation, cell division, gametogenesis, and biological rhythms. Under the catalytic regulation of relevant enzymes, m^6^A participates in various diseases, including tumors, obesity, and infertility (Jiang et al., 2021).

Knocking out the m^6^A demethylase gene *ALKBH5* accelerates the nuclear export of mRNA (Zheng et al., 2013), whereas RNAi silencing of the core of the m^6^A methyltransferase complex METTL3 inhibits the nuclear transport process (Fustin et al., 2013), thereby demonstrating that m^6^A modification can promote nuclear transfer. The nuclear export of mRNA is a bridge connecting mRNA processing in the nucleus and translation in the cytoplasm. Further, nuclear export is coupled with each step of pre-mRNA processing, and only properly processed mRNA is nucleated and translated into protein.

As an exocyclic ring amine involved in Watson Crick base pairing, the rotation direction of Watson Crick base pairing of m6A is opposite to the U force of the carbon-nitrogen bond, showing the methyl group in the reverse conformation, which destabilizes the RNA double strand to local unstructured transcripts (Roost et al., 2015). m6A tends to weaken these structures. This modification is enriched in the 3′-untranslated region (UTR) and alternative splicing exons and introns and is related to mRNA preprocessing, such as splicing regulation and polyadenylation (Meyer et al., 2012). Structural switches, including helices with buried binding sites, can be refolded to allow access to their respective protein partners (Liu et al., 2015). The formation of hybrid structures, such as miRNA-target interactions, is also affected by m^6^A (Brummer et al., 2017).

### 2.2 5-methylcytosine (m^5^C)

**FIGURE 3:**
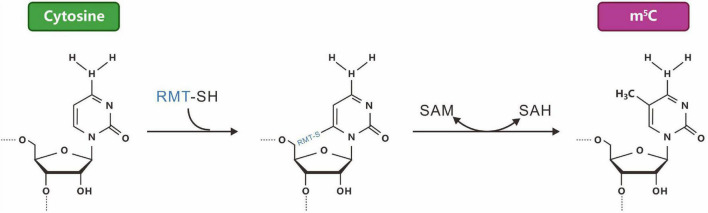
The reaction mechanism of a typical m^5^C-RNA cytosine methyltransferase [m(5)C-RMTs, in blue]. M^5^C, formed by the methylation of carbon 5 of cytosine.

RNA m^5^C modifications were discovered more than 40 years ago. Similar to m^6^A, RNA m^5^C modifications are dynamically reversible. Methyltransferase uses S-adenosyl methionine (SAM) as a methyl donor to methylate cytosine (C) to form m^5^C (Figure 3). The distribution and function of m^5^C may be species- and tissue-specific. Bisulfite treatment combined with transcriptome sequencing has identified m^5^C modification sites in thousands of mRNAs in HeLa cells (Squires et al., 2012). m^5^C antibody immunoprecipitation combined with bisulfite sequencing allowed the identification of multiple m^5^C modifications in archaeal mRNA. The conserved sequence of AU (m^5^C) GANGU was consistent with the m^5^C conserved sequence in archaeal rRNA (Edelheit et al., 2013). The discovery of the m^5^C methyltransferase NSUN2 (NOP2/Sun RNA methyltransferase family member 2) and the binding protein ALYREF (Aly/REF export factor) also proved that RNA m^5^C modifications have dynamic reversibility (Yang et al., 2017b). RNA m^5^C methylation is widespread in cells and plays an important role in various physiological (Flores et al., 2017) and pathological processes, such as tumors (Huang et al., 2021; Nombela et al., 2021), neurological disorders (Van Haute et al., 2019), viral infections (Wnuk et al., 2020), and organism ontogeny (Flores et al., 2017). However, research on RNA m^5^C methylation is still in its infancy.

RNA m^5^C modification can be recognized by the transporter linker protein ALYREF, which promotes the transport of related mRNA out of the nucleus. Knockout of the m^5^C-modified methyltransferase gene *NSUN2* blocks the shuttling of the ALYREF protein between the nucleus and cytoplasm (Yang et al., 2017b). In addition, the patterns of m^5^C distribution on mRNA in relation to *cis*-acting regulatory motifs and miRNA/RISC-binding sites suggest that this modification may be involved in the post-transcriptional regulation of mRNA metabolism (Squires et al., 2012). NSUN2-mediated methylation is required to process non-coding vault RNAs (vtRNAs) into small vault RNAs (svRNAs); however, consequences in the downstream coding transcripts have not emerged because of the defect of RNA m^5^C modification (Hussain et al., 2013). ALYREF can recognize m^5^C in mRNA *via* a methyl-specific RNA-binding motif and regulate the export of bound transcripts in an NSUN2-dependent manner (Yang et al., 2017b), whereas hm^5^C, derived from the Tet-dependent oxidation of m^6^C, preferentially marks mRNAs within coding regions and favors the translation of *Drosophila* transcripts (Fu et al., 2014; Delatte et al., 2016).

### 2.3 Other RNA methylation/modifications

In addition to m^6^A and m^5^C, other RNA modifications, including m^1^A, m^6^Am, hm^5^C, and pseudouridine (Ψ), have been a hot field of research in recent years.

#### 2.3.1 *N*^1^-methyladenosine (m^1^A)

RNA m^1^A methylation was first discovered in non-coding RNAs such as rRNA and tRNA and is widely present in prokaryotic and eukaryotic mRNAs. Unlike m^6^A methylation, m^1^A methylation occurs at the *N*^1^ position of the adenosine base group. It carries a positive charge by blocking Watson-Crick base-pairing under physiological conditions. Thus, it can drastically alter protein-RNA interactions and RNA secondary structures through electrostatic effects. m^1^A maps uniquely to positions near the translation start and first splice sites in coding transcripts and correlates with the upregulation of translation (Dominissini et al., 2016). m^1^A can be removed by ALKBH3 and is responsive to various types of cellular stress (Li et al., 2015a). However, the methyltransferases and binding proteins of m^1^A remain unknown; hence, its specific function and mechanism of action need to be further explored.

In mRNA, m^1^A exists in the highly structured 5′UTRs, indicating that it may change the predicted secondary structure (Dominissini et al., 2016; Li et al., 2016c). In the loop structure, this positive charge may stabilize interactions with the RNA phosphate backbone. The methylation level of m^1^A in transcripts is related to increased translation, which may be due to the availability or direct recruitment of initiation factors and extension factors. The positive charge of this modification enables it to adapt to specific protein RNA and unique RNA-RNA interactions, and its biological effects are still unclear.

#### 2.3.2 *N*^6^, 2′-*O*-dimethyladenosine (m^6^A*^m^*)

The *N*^6^, 2′-*O*-dimethyladenosine (m^6^A*^m^*) modification was found at the first nucleotide of certain mRNAs (Crain et al., 1978). m^6^A*^m^* is formed by the combination of 2′-*O*-methyltransferase (2′-*O*-MTase) and 2′-*O*-methyladenosine-N^6^-methyltransferase. Adenosine is methylated by 2′-*O*-methyltransferase (2′-*O*-MTase) to form A*^m^*, which can then be methylated by 2′-*O*-methyladenosine-*N*6-methyltransferase to form m^6^A*^m^* (Wei et al., 1975). Compared to m^6^A, the m^6^A*^m^* level in RNA is very low. Studies in H1-ESCs and GM12878 cells found that H1-ESC poly(A) + RNAs contained approximately three m^6^A nucleotides per 10^5^ nucleotides compared to ∼100 m^6^A nucleotides per 105 nucleotides, revealing 33 times more m^6^A compared to m^6^A*^m^* (Molinie et al., 2016). m^6^A*^m^* and m^6^A share similar chemical characteristics; m^6^A*^m^* can be detected by m^6^A-seq, and it can be demethylated by FTO and is involved in the regulation of mRNA stability (Mauer et al., 2016). Chen et al. (2020a) demonstrated that METTL4 mediates the *N*6-methylating process of Am30 on U2 small nuclear RNA (snRNA) under an AAG motif *in vitro* and *in vivo* (Chen et al., 2020a). However, the binding protein of m^6^A*^m^* and molecular mechanisms involved in regulating its biological functions require further study.

#### 2.3.3 5-hydroxymethylcytosine (hm^5^C)

**FIGURE 4:**
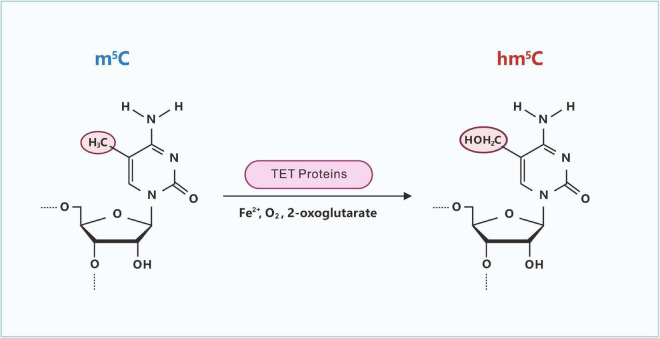
Ten-eleven translocation (Tet)-catalyzed formation of hm^5^C in RNA. The Tet family of Fe(II)- and 2-oxoglutarate-dependent dioxygenases can induce the oxidation of m^5^C to yield hm^5^C.

Similar to m^5^C in DNA, m^5^C in RNA can be oxidized by ten-eleven translocation (Tet)-family enzymes to hm^5^C (Figure 4; Fu et al., 2014). The Tet family of Fe(II)- and 2-oxoglutarate-dependent dioxygenases can induce the oxidation of m^5^C to yield hm^5^C (Kriaucionis and Heintz, 2009; Ito et al., 2011). In *Drosophila melanogaster*, which lacks DNA hydroxymethylation, hm^5^C is present in greater than 1,500 mRNAs, and hm^5^C modification is mainly distributed in the exons of mRNA and contains CU-enriched conserved motifs (Delatte et al., 2016). hm^5^C modification can promote the translation efficiency of mRNA, which is significantly higher than that of RNA without hm^5^C modifications. After *TET* knockout, the level of hm^5^C modification in RNA is reduced, resulting in the abnormal development of the *Drosophila* brain (Delatte et al., 2016).

#### 2.3.4 Pseudouridine (Ψ)

**FIGURE 5:**
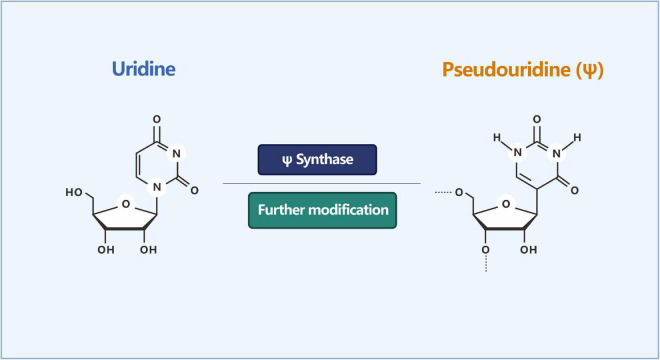
Isomerization reaction of uridine into pseudouridine (Ψ). ψ is formed by the sequence-specific isomerization of uracil (U).

In addition to methylated modifications, there are some unmethylated modifications of RNA, such as ψ (sometimes referred to as pseudouracil). It is one of the most abundant forms of post-transcriptional RNA modifications, widely present in cellular RNA, and highly conserved among species. ψ is formed by the sequence-specific isomerization of uracil (U) (Figure 5), which is abundant in tRNA and rRNA (Goodman et al., 1968). Ψ is known to affect the secondary structure of RNA, and its function in altering stop codon read-through may also be biologically relevant (Karijolich and Yu, 2010; Fernandez et al., 2013).

## 3 Dynamic regulation of RNA methylation/modifications

### 3.1 Methyltransferase-writers

#### 3.1.1 Core m^6^A methyltransferase complex components: METTL3, METTL14, and WTAP

**FIGURE 6:**
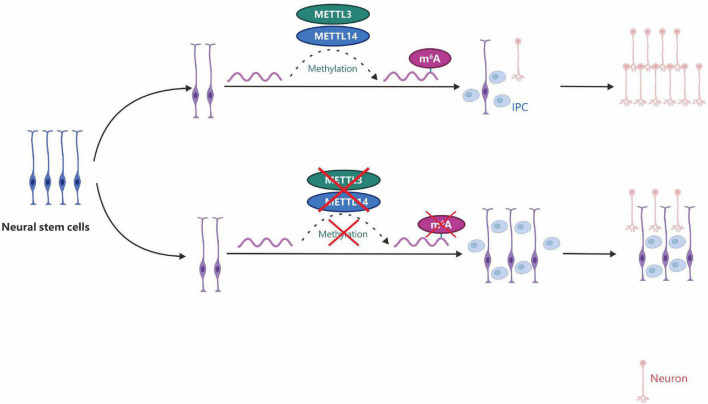
m^6^A depletion by *METTL3*/*14* gene knockdown can promote the proliferation of neural stem cells and lead to prolonged cell cycle progression and maintenance of radial glial cells. IPC, intermediate progenitor cells.

The RNA m^6^A methyltransferase holoenzyme complex consists of at least two multicomponent factors of the whole methyltransferase called MT-A (∼ 200 kDa) and MT-B (∼ 800 kDa). However, only the METTL3 (methyltransferase like 3, ∼ 70 kDa) protein has been identified, and the subunit alone had no enzymatic activity (Tuck, 1992). METTL3 is widely present in various human tissues, especially in the testes (Leach and Tuck, 2001). METTL3 has two key domains that are used to combine SAM and catalyze the formation of m^6^A (Figure 6). Knocking down *METTL3* causes apoptosis in human HeLa and HepG2 cells, accompanied by a significant decrease in m^6^A levels (Dominissini et al., 2012). Apart from the METTL3 component, other components of the methyltransferase complex have not been comprehensively studied. In 2014, an evolutionary analysis of the METTL3 family revealed the *METTL14* and *METTL4* genes, which are highly homologous to different families of *METTL3* (Bujnicki et al., 2002). METTL14 is another component in the m^6^A methyltransferase complex with enzymatic activity (Liu et al., 2013). In HeLa and HEK 293FT cells, knocking down *METTL14* resulted in a decrease in total mRNA of m^6^A content, while METTL14 and METTL3 interacted with each other. *In vitro* size exclusion chromatography (gel filtration) experiments and two-dimensional gel electrophoresis analysis showed that METTL14 and METTL3 can form a stable complex at a 1:1 ratio. *In vitro* experiments on the activity of enzymes involved in m^6^A formation showed that although a single METTL14 had slightly higher enzymatic activity than METTL3, the heterodimer formed by METTL14 and METTL3 with a strong preference for substrates had the highest enzymatic activity for the RNA of the stem-loop structure, which has no obvious secondary structures. It preferentially methylates GGACU, which is consistent with the conserved sequence of m^6^A distribution reported previously (Liu et al., 2013).

The third component of the mammalian m^6^A methyltransferase complex is WTAP (Ping et al., 2014b). WTAP plays a key role in transcriptional and post-transcriptional regulation and is an oncogene whose expression is elevated in several types of human tumors. Through a yeast two-hybrid experiment, FIP37 (FKBP12 interacting protein 37, At3g54170, which encodes a human WTAP homologous gene) was found in Arabidopsis as a protein that interacts with methylthioadenosine (MTA) (Zhong et al., 2008). In HeLa cells, WTAP can co-precipitate with the METTL3-METTL14 heterodimer, and their combined effect is weaker than the interaction between METTL3 and METTL14 (Liu et al., 2013). WTAP lacks a catalytic region and has no methyltransferase activity, but the interaction between WTAP and METTL3-METTL14 can localize the methyltransferase complex in nuclear spots (Ping et al., 2014a), thereby regulating the binding of the methyltransferase complex to the target RNA, which ultimately affects the m^6^A level. Downregulating WTAP can suppress the localization of METTL3 in the nucleus and reduce m^6^A levels. The field has demonstrated that the consistency of the RNA substrates of the WTAP, METTL3, and METTL14 combination reached 36% using immunoprecipitation (PAR-CLIP) experiments (Liu et al., 2013). The conserved sequence of RNA binding is the same as the previously reported conserved sequence of m^6^A, and the three sites of RNA binding are mainly located in the intergenic and intron regions, indicating that m^6^A is generated from the precursor RNA. Silencing of the methyltransferase complex increases the expression of bound RNA, indicating that m^6^A is inversely related to gene expression (Batista et al., 2014; Schwartz et al., 2014a). Because WTAP is a splicing factor, knocking down *WTAP* or *METTL3* can result in the formation of different isoforms of RNA containing m^6^A (Dominissini et al., 2012; Ping et al., 2014a), indicating that m^6^A affects alternative splicing of RNA.

Taken together, the discovery of METTL3, METTL14, and WTAP, which are important active center components in the m^6^A methyltransferase complex (Yao et al., 2020), has provided many new insights into m^6^A, laying an important foundation for revealing the biological function of m^6^A in RNA.

#### 3.1.2 Other m^6^A methyltransferase complex components

##### 3.1.2.1 KIAA1429

KIAA1429 is an important methyltransferase that participates in m^6^A modifications (Schwartz et al., 2014b). Based on the protein immunoprecipitation-mass spectrometry analysis of the core component of m^6^A methyltransferase, KIAA1429 may be a new subunit component of the m^6^A methyltransferase complex to catalyze the formation of m^6^A in mRNA (Schwartz et al., 2014). In *Drosophila*, the homologous gene of KIAA1429 interacts with the homologous gene of WTAP and regulates the pre-mRNA selective splicing of the important gene sex lethal (*Sxl*), which controls sex determination (Ortega et al., 2003). In human A549 cells, deletion of KIAA1429 can cause a sharp drop in the m^6^A peak, indicating that KIAA1429 plays an important role in the methyltransferase complex (Schwartz et al., 2014). Another study found that KIAA1429 can recruit METTL3/METTL14/WTAP, the catalytic core of the m^6^A methyltransferase complex, to achieve site-specific regulation of m^6^A levels in mRNA (Yue et al., 2018).

##### 3.1.2.2 RNA binding motif protein 15(B) (RBM15/RBM15B)

*RBM15*/*15B*, a protein-coding gene, is a part of the WTAP-METTL3 m^6^A methyltransferase complex that interacts with WTAP to recruit the complex to target mRNAs. RBM15 and RBM15B can participate in catalyzing the formation of m^6^A on mRNA and non-coding RNA X-inactive specific transcripts (XIST). Further, immunoprecipitation experiments showed that RBM15 and RBM15B could bind and recruit the WTAP-METTL3 complex to specific target sites. RBM15/RBM15B consists of an RNA recognition domain, as per iCLIP (individual nucleotide resolution crosslinking and immunoprecipitation) sequencing and single miCLIP (m^6^A individual nucleotide resolution crosslinking and immunoprecipitation). Sequencing analysis of the base resolution revealed that the RBM15/RBM15B-binding site was significantly enriched near the m^6^A methylation modification site (Patil et al., 2016). In addition, the homologous protein Nito of RBM15 was also identified as a component of the methylase complex in *Drosophila* (Lence et al., 2016).

##### 3.1.2.3 METTL16

**FIGURE 7:**
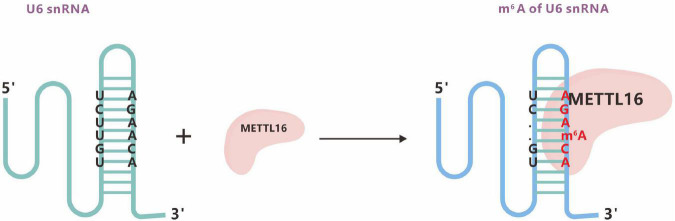
The m^6^A methyltransferase METTL16 binds U6 snRNA. m^6^A typically locates at a DRACH motif, where D denotes A, G, or U; R denotes A or G; and H denotes A, C, or U.

METTL16, a homologous protein of METTL3, can mediate the formation of m^6^A in U6 snRNA. A 2017 study showed that METTL16 could also mediate the methylation of mRNA and regulate the intracellular levels of SAM. METTL16 contains methylation marks within the conserved UACm^6^AGAGAA sequence in the 3′UTR hairpins of the mRNA MAT2A and a special type of RNA structure (Figure 7; Pendleton et al., 2017).

##### 3.1.2.4 Zinc finger CCCH-type containing 13 (Zc3h13)

**FIGURE 8:**
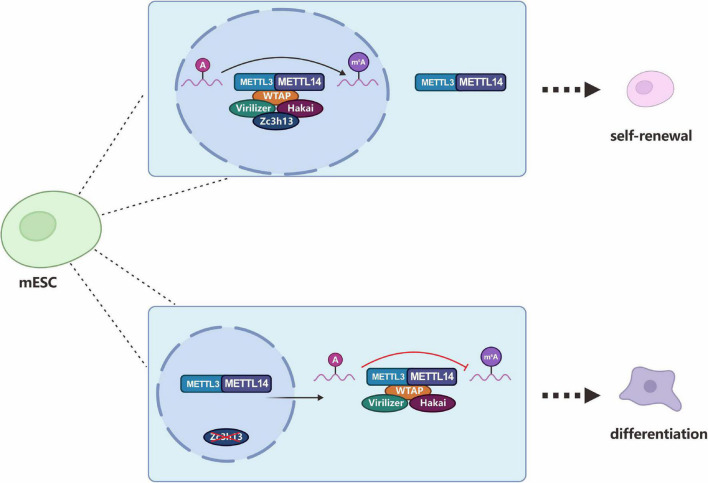
Zc3h13 modulates RNA m^6^A methylation in the nucleus. Zc3h13 anchors WTAP, Virilizer, and Hakai in the nucleus to facilitate m^6^A methylation and to regulate mESC self-renewal. Upon Zc3h13 knockdown, a great majority of WTAP, Virilizer, and Hakai translocate to the cytoplasm to inhibit m^6^A methylation.

Zc3h13, a critical RNA m^6^A regulator, plays an important role in modulating RNA m^6^A methylation in the nucleus (Wen et al., 2018). In 2018, three studies almost simultaneously described Zc3h13 as a new component of the m^6^A methyltransferase complex that regulates m^6^A modification. Wen et al. (2018) found that mouse Zc3h13 can stabilize the nuclear localization of the Zc3h13-WTAP-Virilizer-Hakai complex and regulate the self-renewal of mouse embryonic stem cells (mESCs) by promoting m^6^A methylation (Figure 8). Knuckles et al. (2018) further clarified that mouse Zc3h13 and its *Drosophila* homologous gene *CG7358* (named *Flacc*) mediate the interaction between RBM15/Nito and WTAP/Fl (2)d, thereby promoting m^6^A modification of Mrna. They also found that *Flacc* regulates sex determination and dosage compensation in *Drosophila* by regulating alternative splicing of the *Sxl* gene. Guo et al. (2018) identified the *CG7358* gene of *Drosophila* (named *Xio*) and its involvement in the sex determination pathway of *Drosophila* through regulating the alternative splicing of *Sxl via* m^6^A modification.

In addition to the above-mentioned members, there are other methyltransferase components involved in the selective recognition of methylation sites and division of labor to ensure fine post-transcriptional regulation.

#### 3.1.3 m^5^C methyltransferase: NSUN2 and DNMT2

**FIGURE 9:**
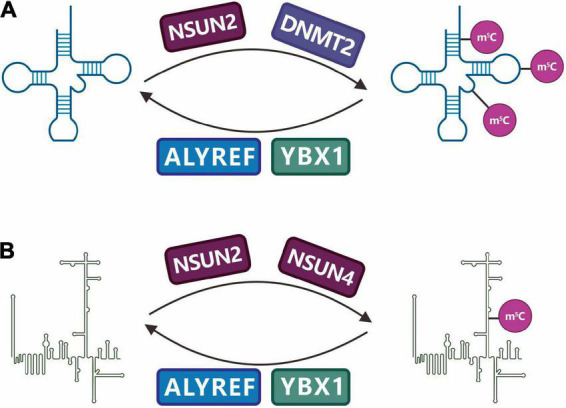
Regulation and function of RNA m^5^C methylation. C5-methylcytidine (m^5^C) is a common modification in **(A)** tRNAs and **(B)** other non-coding RNAs (ncRNAs). NSUN family enzymes (NSUN2, NSUN4) and DNMT2 have been identified as m5C writers, while ALYREF and YBX1 have been identified as m5C readers.

The identified RNA m^5^C-specific methyltransferases include the NSUN (NOL1/NOP2/SUN) family proteins and DNMT2 (DNA methyltransferase 2), which belong to the superfamily of Rosman-folded enzymes and are characterized by the conserved cysteine residue SAM. The RNA m^5^C methyltransferase that has been studied the most recently is the NSUN family, which acts as a methyl donor to catalyze the transfer of methyl groups to cytosine residues of different RNA substrates (Bujnicki et al., 2004). Among the nine members of this family, many have catalytic and release sites for methyltransferases, particularly NSUN2 (also known as Trm4). NSUN2, encoded by the NSUN2 gene on chromosome 5p15.31-33, is a nucleolar RNA methyltransferase that catalyzes the m^5^C methylation of various RNAs such as mRNA, tRNA, and ncRNA (Figure 9). Ultrahigh-performance liquid chromatography-triple quadrupole mass spectrometry with multiple-reaction monitoring (UHPLC-QQQ-MRM-MS/MS) studies have found that NSUN2 is an mRNA-specific m^5^C methyltransferase, and its catalytic activity depends on the C271 (cysteine 271) and C321 (cysteine 321) sites, where C321 catalyzes the methylation of cytosine by binding to the cytosine pyrimidine ring to form a covalent bond, and C271 mediates the release of RNA. High-throughput sequencing analysis also showed that *NSUN2* knockdown significantly reduced mRNA m^5^C modification (Yang et al., 2017b).

In addition to NSUN2, DNMT2 is an RNA m^5^C methyltransferase that plays a role in multiple species. It was initially considered a DNA methyltransferase, but the latest research shows that human DNMT2 cannot catalyze DNA methylation but can catalyze miRNA methylation and the changes in the C38 methyl group of tRNA*^Asp^* (aspartatetRNA) (Figure 9; Goll et al., 2006).

### 3.2 Demethylase-erasers

#### 3.2.1 m^6^A demethylase: FTO and ALKBH5

During brain development, a dynamic change in m^6^A levels in RNA has been observed (Meyer et al., 2012), suggesting the existence of RNA m^6^A demethylases. Two novel mRNA m^6^A demethylases (FTO and ALKBH5) have recently been identified, confirming the dynamic regulation of m^6^A (Jia et al., 2011; Zheng et al., 2013).

The *FTO* gene is a major regulator of metabolism and energy utilization (Church et al., 2009, 2010; Fischer et al., 2009). It is a member of the Fe(II)- and oxoglutarate-dependent AlkB oxygenase family and was originally shown to catalyze the oxidative demethylation of methylated thymidine and uracil (Gerken et al., 2007; Jia et al., 2008). Overexpressing FTO in mice causes excessive energy intake and fattening; knocking out *FTO* makes mice thinner and grow slowly and causes teratogenic and lethal phenomena. In addition, FTO is closely related to diseases such as type 2 diabetes, cancer, and dementia (Boissel et al., 2009; Fu et al., 2013; Zhao et al., 2014). FTO can remove ^3^mT and m^3^U from single-stranded DNA and RNA (Jia et al., 2008), but its activity is very weak, and these nucleic acid modifications are rarely found in the body. It is generally believed that these are not the actual substrates for FTO. In 2011, m^6^A was reported to be an enzyme substrate for FTO (Jia et al., 2011). By simulating the demethylation reaction conditions in the physiological environment *in vitro*, the wild-type and mutant proteins of the obesity gene *FTO* were incubated with methylated substrates, and various methylation forms were analyzed using mass spectrometry and high-performance liquid phase technology. FTO had a demethylation function for m^6^A on single-stranded RNA, and the enzyme kinetic data indicated that the activity of FTO on m^6^A was much higher than that on m^3^U. *In vivo* experiments showed that the m^6^A content of mRNA in cells with knockdown of the *FTO* gene increased by approximately 23%; overexpression of FTO reduced the m^6^A content of mRNA by approximately 18% (Jia et al., 2011). Both *in vivo* and *in vitro* data indicate that the actual demethylase substrate of FTO is m^6^A on nuclear RNA. MeRIP-seq analysis of *FTO*-knockout brain tissue showed that the m^6^A level was almost unaffected, but the m^6^A level of certain specific mRNAs was significantly increased, indicating that FTO acts only on specific mRNA substrates (Hess et al., 2013). Epidemiological fluorescence experiments indicated that FTO is partially located in nuclear spots, suggesting that nuclear RNA is the main substrate of FTO (Jia et al., 2011).

Soon after FTO was discovered, a study reported a second m^6^A demethylase, ALKBH5, in mammals (Zheng et al., 2013). Integrating mass spectrometry, cell biology, genomics, bioinformatics, and model biology revealed the catalytic m^6^A demethylation activity of ALKBH5 (RNA demethylase). As the second identified m^6^A demethylase, ALKBH5 has m^6^A demethylase activity comparable to that of FTO and preferentially acts on the m^6^A site of conserved motifs. Unlike the oxidative demethylation of FTO, ALKBH5 can directly catalyze the removal of m^6^A without generating intermediate products (Zheng et al., 2013). In addition to FTO and ALKBH5, more m^6^A demethylases are yet to be discovered.

### 3.3 Binding proteins-readers

#### 3.3.1 m^6^A readers

The discovery of m^6^A methyltransferase and demethylase in RNA means that m^6^A is a reversible chemical modification that can dynamically regulate physiological development processes. It is currently known that m^6^A-binding proteins in mammals are mainly of families containing YTH (YT521-B homology) domains, including YTHDF1, YTHDF2, YTHDF3, YTHDC1, YTHDC2, and heterogeneous nuclear ribonucleoproteins (HNRNP). Readers mainly work by directly or indirectly binding RNA. Direct RNA binding is mediated by a reader-specific recognition modification domain such as the YTH family proteins, including YTHDF1, YTHDF2, YTHDF3, YTHDC1, and YTHDC2, which can directly bind to methylation modifications of the target genes in the nucleus and cytoplasm (Figure 2). It can affect mRNA modifications, including mRNA splicing, nuclear export, translation, and mRNA degradation (Nachtergaele and He, 2017; Zhao et al., 2017). Indirect RNA binding relies on methylation-induced RNA unwinding, thereby exposing hidden protein-binding motifs and making proteins more likely to bind to RNA. HNRNP, known as heterogeneous nuclear ribonucleoproteins and indirect RNA-binding proteins, is a complex of proteins and RNA found in the nucleus of cells during gene transcription and subsequent post-transcriptional modification of newly synthesized RNA. It is involved in various cellular functions, including mRNA stability (Zhao et al., 2009), mRNA transport (Ma et al., 2018), miRNA maturation (Guil and Cáceres, 2007), and telomere biogenesis (Labranche et al., 1998).

##### 3.3.1.1 YT521-B homology domain family members

YTH is a newly discovered domain that can bind to short, denatured single-stranded RNA (Zhang et al., 2010). The human genome contains at least five YTH domain-containing proteins: YTHDF1–3 and YTHDC1–2. Some YTH proteins contain a low-complexity region (LCR) that can form a phase separation of the protein (Ries et al., 2019), causing the cytoplasmic YTHDF-m^6^A-RNA polymer to become processing bodies (P-bodies). P-bodies are cytoplasmic ribonucleoprotein (RNP) granules, which are aggregates of multiple functional proteins and RNA in the cytoplasm (Wang et al., 2014). The binding capacity of the YTH domain to RNA containing conserved m^6^A sequences is approximately 1 μmol/L (Wang et al., 2014; Xu et al., 2014, 2015; Zhu et al., 2014).

In 2014, the m^6^A-binding protein YTHDF2 (YTH domain family, member 2) was first reported to mediate the degradation of m^6^A-modified mRNA (Wang et al., 2014). Photoactivatable ribonucleoside–enhanced crosslinking and PAR-CLIP sequencing results indicated that YTHDF2 mainly binds to mRNA and some long non-coding RNA, and the binding site is mainly in the 3′UTR rich in GAC sequences, which is very similar to the distribution characteristics of m^6^A. In general, YTHDF2 colocalizes with the deadenylation and decapsidase complex in the cell, which can direct the RNA substrate to the cytoplasmic subcellular organelle P-body that mediates RNA degradation (Wang et al., 2014). Subsequent studies further showed that YTHDF2 accelerated the degradation of m^6^A-modified transcripts by recruiting CCR4-NOT adenylate complexes (Du et al., 2016), primarily comprising subunits with deadenylase activity (CNOT6, CNOT6 L, CNOT7, or CNOT8) and regulatory NOT modules (CNOT1, CNOT2, CNOT3, CNOT9, CNOT10, and CNOT11).

Similar to YTHDF2, the second identified m^6^A-binding protein is YTHDF1 (YTH domain family, member 1), which is also a cytoplasmic protein that can promote the translation efficiency of the m^6^A-mRNA it binds. Ribosomal map analysis showed that YTHDF1 promotes the binding of substrate RNA to ribosomes, which is conducive to RNA translation (Wang et al., 2015). In addition, YTHDF1 promotes translation initiation, which, in turn, promotes protein synthesis. Downregulation of YTHDF1 will result in a reduction in substrate RNA translation efficiency, and the magnitude of the reduction is directly related to how much YTHDF1 is downregulated. The discovery of the YTHDF1 function indicates that m^6^A, as a dynamic RNA marker, can effectively regulate protein production.

YTHDC1, an m^6^A-binding protein in the nucleus, recognizes and binds m^6^A-containing RNAs and can mediate m^6^A-regulated mRNA splicing (Xiao et al., 2016). YTHDC1 is located at the YT body of the nucleus. *In vitro* electrophoretic mobility shift assay (EMSA) experiments showed significant binding of YTHDC1 to RNA containing m^6^A modifications, while *in vivo* photoactivatable ribonucleoside enhanced crosslinking and PAR-CLIP sequencing showed that the binding motif of YTHDC1 is consistent with the conserved motif RRACH of m^6^A. Most of the binding sites were located near the stop codon, which is also consistent with the distribution characteristics of m^6^A. YTHDC1 can interact with the splicing factor SR protein to regulate the alternative splicing of m^6^A-containing exons (Xiao et al., 2016). YTHDC1 also promotes XIST-mediated X-chromosome silencing by identifying the m^6^A modification site on the non-coding gene *XIST* (Patil et al., 2016). In HeLa cells, YTHDC1 interacts with SRSF3 and the RNA export factor 1 (NXF1) to promote the export of m^6^A-modified mRNA (Roundtree et al., 2017b).

YTHDF3 interacts with YTHDF1 and YTHDF2 to enhance the binding ability of YTHDF1 or YTHDF2 to RNA containing an m^6^A-modified substrate, thereby promoting RNA translation or degradation. YTHDF3 may also interact with other proteins to exert cell-specific regulatory functions (Li et al., 2017; Ni et al., 2019).

YTHDC2 enhances the translation efficiency of target mRNA by binding to the m^6^A-conserved motif (Wojtas et al., 2017). YTHDC2 also plays an important role in spermatogenesis by interacting with the meiosis-specific protein MEIOC to affect substrate stability (Abby et al., 2016; Soh et al., 2017). A study using infertile male and female mice with *YTHDC2* knockout presented a defective phenotype during meiosis phase I (Tanabe et al., 2016). In addition to containing the YTH domain, YTHDC2 also contains an RNA-binding domain, a helicase domain, and two ankyrin repeats, which may play a role in the recruitment of RNA secondary structures, RNA binding proteins, and other interacting proteins (Abby et al., 2016; Tanabe et al., 2016). This also suggests that YTHDC2 may play multiple roles in various biological processes.

##### 3.3.1.2 HNRNP family members

The HNRNP protein family is mainly distributed in the nucleus and is an RNA-binding protein that participates in processes such as precursor RNA splicing, transportation, and translation (Han et al., 2010). HNRNPA2B1, a member of the HNRNP family, can bind m^6^A-modified RNA transcripts both *in vivo* and *in vitro*. However, it remains controversial whether HNRNPA2B1 is an m^6^A-binding protein. Alarcón et al. (2015) found that HNRNPA2B1 can directly bind to m^6^A modification sites and regulate alternative splicing and pri miRNA processing, while Wu et al. (2018) found that HNRNPA2B1 does not directly bind m^6^A but acts as a converter based on structural studies. In addition, two other HNRNP family proteins, HNRNPC and HNRNPG, can regulate the processing of RNA transcripts containing m^6^A modifications. Unlike HNRNPA2B1, HNRNPC and HNRNPG do not directly bind to the m^6^A site but mediate the alternative splicing process of transcripts containing m^6^A by recognizing and binding m^6^A-dependent structural switches (Liu et al., 2015, 2017).

Additionally, studies based on RNA pull-down experiments have detected other potential m^6^A binding proteins, including ELAV-like RNA binding protein 1 (ELAVL1, also called HuR), FMRP translational regulator 1 (FMR1), leucine-rich pentatricopeptide repeat containing (LRPPRC), and insulin-like growth factor 2 mRNA-binding proteins (IGF2BPs).

#### 3.3.2 m^5^C-binding proteins: ALYREF

To identify the binding protein of m^5^C, two oligonucleotide substrates, with and without m^5^C, were designed. Through oligonucleotide enrichment combined with protein profiling, the mRNA was identified as a nuclear functional complex component. ALYREF binds to m^5^C-modified RNA oligonucleotides. EMSA and RIP combined with HPLC and sequencing technology demonstrated that ALYREF’s K171 (lysine 171) mutation can significantly reduce its ability to bind m^5^C, thereby reducing its ability to bind RNA. Further, fluorescence *in situ* hybridization technology (FISH) showed that the efficiency of mRNA nucleation decreased with the knockdown of *NSUN*2 and *ALYREF*. However, backfilling experiments demonstrated that only the wild-type NSUN2 and ALYREF could reverse the reduction in mRNA nucleation caused by the knockdown of *NSUN2* and *ALYREF*, suggesting that m^5^C plays an important regulatory role in the process of mRNA nucleation (Shi et al., 2017; Yang et al., 2017a; Fan et al., 2019).

## 4 Biological processing of RNA methylation

### 4.1 m^6^A and neural stem cells

m^6^A is distributed in distinct developmental stages in the brain and controls the self-renewal of neural stem cells (NSCs) and their differentiation into neurons (Yoon et al., 2017; Du et al., 2019; Zhu et al., 2020). In *METTL3*/*METTL14* gene knockout mice, m^6^A can promote the proliferation of NSCs and prolong the cell cycle of radial glial cells (Figure 6; Yoon et al., 2017; Wang et al., 2018b). Such regulation may promote the development of stem cell and gene-targeted therapies for Alzheimer’s, Parkinson’s, and cognitive-related neurological diseases.

### 4.2 m^6^A and synaptic functions

m^6^A modifications also contribute to the local regulation of synaptic functions (Flamand and Meyer, 2019). Experimental studies have shown that FTO protein in neurons can shift between the nucleus, cell body, and dendrites (including synapses) and can lead to changes in local RNA methylation kinetics (Meyer et al., 2012; Chang et al., 2017). Methylated transcripts are highly biased toward neuronal genes and functions such as synaptic functions. The m^6^A transcriptome is spatially regulated in different brain regions. At the single neuron level, m^6^A-modified RNA and its interaction groups diffuse to specific structures such as axons, dendrites, pre-synaptic nerve endings, and dendritic spines. This spatial distribution supports the m^6^A’s functional library to control synapses, touch transmission, and plasticity. Transcriptomic analysis of m^6^A in a mammalian brain revealed a specific bias of m^6^A toward neuronal genes rather than glial cell genes. The functional classification of m^6^A-seq analysis in the entire mouse brain, midbrain, cortex, and cerebellum has been identified. Most m^6^A-target genes are involved in nervous system development, synaptic transmission, and post-synaptic function. Other studies have reported the localization of m^6^A in axons and its role in axon growth. Transcripts encoding the axon elongation factor growth-associated protein-43 (GAP-43), a specific marker for axonal regeneration, were identified as targets for m^6^A modifications. The local translation of these transcripts is negatively regulated by m^6^A and can be regulated by FTO in axons (Geuens et al., 2016; Yu et al., 2018), showing that RNA methylation also plays an important role in the development and function of synapses.

### 4.3 m^6^A regulates nervous system development

Among the various tissues and organs, the brain has the most abundant expression of m^6^A and its recognition proteins. m^6^A-related regulatory proteins perform important functions in the cerebral cortex, such as synaptic function, axon regeneration, neural stem cell self-renewal, and cerebellar development (Yoon et al., 2017). The m^6^A modification plays an irreplaceable regulatory role in the development and function of the nervous system. *Drosophila* with a knockout of a yeast m^6^A methyltransferase encoded by the *Ime4* gene can be born and survive until adulthood; however, the life span of the fruit fly is shortened and shows obvious abnormal behavior, suggesting that the deletion of m^6^A modification affects the function of the fruit fly nervous system (Lence et al., 2016). A specific knockout of *METTL14* in the central nervous system of mice can affect the development of the mouse cerebral cortex (Yoon et al., 2017). Moreover, the deletion of the *YTHDF2* gene in mice leads to an increase in the overall level of m^6^A, preventing the differentiation of neural stem cells and the formation of neuronal axis dendrites from entering the RNA degradation pathway, resulting in the asymmetric division of neural stem cells in the cerebral cortex (Li et al., 2018b). When neural precursor cells are missing in mice, the differentiation of neurons is affected, resulting in the slower development of the forebrain cortex.

Except for the cerebral cortex, cerebellar RNA m^6^A patterns and levels are particularly prominent. The dynamic process of m^6^A methylation and demethylation occurs throughout the entire developmental process of the cerebellum after birth, and the lack of *ALKBH5* in a low-pressure and low-oxygen environment causes the m^6^A level of genes involved in the regulation of cerebellar development to be disordered, speeding up the process of RNA export and leading to a marked lag in cerebellar development (Ma et al., 2018). The specific knockout of *METTL3* in the CNS causes severe motor dysfunction in mice during lactation and leads to death. Anatomical and pathological examination revealed that the deletion of m^6^A methylation caused by *METTL3* knockout severely affected the development of the cerebral cortex and cerebellum, resulting in thinning of the cerebral cortex and dysplasia of the cerebellum (Wang et al., 2018a). The absence of m^6^A also causes disordered gene expression regulation during the differentiation and maturation of granule neurons in the cerebellum, resulting in severe apoptosis of newborn granule neurons, revealing that METTL3-mediated m^6^A modification plays an important role in the development of the mammalian central nervous system (Wang et al., 2018a).

### 4.4 m^5^C is involved in brain development

m^5^C-modified methyltransferase NOP2/Sun RNA methyltransferase 2 (NSUN2), a protein-coding gene, also regulates neuronal development. In a developing mouse brain, the absence of NSUN2 did not affect RG cells but delayed their differentiation into superior neurons. NSUN2 is expressed in early neuroectodermal cells and can differentiate into various region-specific neuronal and glial cell types (Li et al., 2005). Mutations or deletions in the m^5^C methylase genes *NSUN2* and *NSUN3* can cause defects in the nervous system. *NSUN2* mutations are associated with autosomal recessive mental retardation (Khan et al., 2012). In the mouse brain, NSUN2 is localized in the nucleolus of Purkinje cells in the cerebellum. The mutated NSUN2 cannot function normally because it cannot converge in the nucleolus. Further research revealed that the methylation level of tRNA in the brains of NSUN2-deficient mice decreased, the cerebral cortex thickened, the number of intermediate progenitors increased, and the number of upper-layer neurons decreased. In this process, the loss of NSUN2 may result in a reduction in the size of the hindbrain due to the failure to produce sufficient numbers of differentiated neurons, while the migration ability of human neuroepithelial stem cells with NSUN2 loss is significantly reduced, which also suggests that NSUN2-dependent tRNA methylation is essential for brain development. The differentiation and migration of neural progenitor cells thus play an important role (Flores et al., 2017). In addition, the inactivation of NSUN3 in mouse embryonic stem cells can further lead to impaired differentiation of the neuroectodermal lineage (Trixl et al., 2018). The summary of RNA modifications is presented in Table 1.

**TABLE 1:** The summary of RNA modifications.

Modification types	RNAs presented	Modifying enzymes and modification readers	Biological function
*N*^6^-methyladenosine (m6A)	mRNAs, rRNAs, ncRNAs, lncRNAs.	Write: METTL3 METTL14 WTAP KIAA1429 RBM15 RBM15B METTL16 Reader: YTHDF1 YTHDF2 YTHDF3 YTHDC1 YTHDC2 eIF3 HNRNPA2B1 HNRNPC Eraser: ALKBH5 FTO	Regulating the transcriptome of eukaryotes and processes such as mRNA stability, splicing, nucleation, localization, and translation.
5-methylcytosine (m5C)	mRNAs, tRNAs, rRNAs, miRNAs, lncRNAs, circRNAs.	Writer: NSUN1 NSUN2 NSUN3 NSUN4 NSUN5 NSUN6 NSUN7 DNMT2 Reader: ALYREF YBX1 Eraser: still under debate	1. Stabilizing the secondary structure. 2. Influencing the anticodon stem-loop conformation. 3. Affecting translational fidelity.
*N*^1^-methyladenosine (m1A)	tRNAs, rRNAs, mRNAs.	Writes: TRMT61A TRMT6 TRMT10C TRMT61B Reader: remaining controversial Eraser: ALKBH3 ALKBH1	1. Facilitating translation. 2. Affect tRNA structure folding. 3. Stabilizing the tertiary structures of the tRNA molecules.
Pseudouridine (Ψ)	rRNAs, tRNAs, snRNAs.	Writes: PUS1 PUSL1 PUS3 PUS7 PUS7L PUS10 RPUSD1 RPUSD2 RPUSD3 RPUSD4 TRUB1 TRUB2 DKC1 Reader: none identified Eraser: none identified	Stabilizing secondary structures to alter translation efficiency, RNA localization, and RNA stability.
*N*^6^, 2′-*O*-dimethyladenosine (m6Am)	mRNAs, rRNAs, snoRNAs	Writes: METTL4 Reader: none identified Eraser: FTO	1. Inhibiting adenosine deamination, increase mRNA stability. 2. Controlling RNA stability and translation.

m6A, *N*^6^-methyladenosine; m5C, 5-methylcytidine; m1A, *N*1-methyladenosine; Ψ, pseudouridine; mRNA, messenger RNA; rRNAs, ribosomal RNA; ncRNA, non-coding RNA; lncRNA, long non-coding RNA; tRNAs, transfer RNAs; miRNA, micro RNA; circRNA, circular RNA; snRNAs, small nuclear RNAs; snoRNAs, small nucleolar RNAs.

## 5 Methods to detect RNA modifications

### 5.1 Wet-lab approaches applied for profiling RNA epitranscriptome

Targeted approaches for detecting RNA modifications have been well established. These include roughly four types (Vandivier and Gregory, 2017): (1) direct sequencing through nucleotide labeling and chromatography, (2) mass spectrometry, (3) detecting the stalling and termination of reverse transcriptase (RT), and (4) direct measurement of changes in base pairing. We have summarized these targeted technologies for detecting specific modifications (Li et al., 2016b; Helm and Motorin, 2017; Lv et al., 2021). Recently, the progress in combining existing biochemical techniques and high-throughput sequencing has been rapid in covalent RNA modification studies. These technologies include: (1) high-throughput sequencing + antibody pull-down and (2) high-throughput sequencing + chemical conversion and chemical adduct coupling methods (Vandivier and Gregory, 2017). (3) Single-molecule real time (SMRT) technology using nanowells and nanopore sequencing (Furlan et al., 2021). Recently developed *in silico* methods that detect RT errors in high-throughput RNA sequencing data and high-throughput single molecule sequencing data may read transcriptome and extratranscriptome information simultaneously (Helm and Motorin, 2017; Vandivier and Gregory, 2017). These methods are summarized in Table 2.

**TABLE 2:** Methods for detecting RNA modifications.

Methods	Detection	Pros	Strengths	Drawbacks
Direct sequencing	*Via* nucleotide labeling and chromatography	Various modifications	● Powerful and able to produce high quality data	● Labor intensive ● Restricting its use to highly abundant RNAs
Direct sequencing with SCARLET	Site-specific cleavage and radioactive-labeling followed by ligation-assisted extraction and thin-layer chromatography	m6A Ψ	● Purifing less abundant species of RNA ● Including sequence information ● Quantification possible ● No specialized equipment	● Single-site query ● No high throughput ● Labor intensive
Direct sequencing with mass spectrometry	Analyze fragmented and whole RNA	Various modifications	● Unbiased manner ● Highly accurate quantification ● Expertise is reasonably widespread	● Labor-intensive ● No sequence information ● Requires specialized equipment ● Methodological and computational challenges
Reverse transcriptase-based methods	Detecting the stalling and termination	Various modifications	● Targeting transcripts in a heterogeneous pool of RNA ● Ideal approach for studying less abundant RNAs ● Straightforward protocol ● Precise single-nucleotide mapping ● Adaptable to different types of modification	● Semi-quantitative
High-resolution melting	DNA polymorphisms DNA methylation covalent RNA modifications	Various modifications	● Performing with any existing set of PCR probes ● Covering a putative modification site	● Putative modifications ● Relying on a shift in melting temperature.
**Global methods for detecting RNA modifications**				
Antibody-based enrichment coupled to high-throughput sequencing	Methyl and hydroxymethyl RIP-seq, which rely upon antibodies recognizing modified ribonucleotide epitopes	m^6^A m^1^A Ψ m^5^C hm^5^C	● Unbiased surveys	● Modification sites cannot be defined with singlenucleotide resolution
High-throughput sequencing with chemical-based methods	Specifically target or exclude modified ribonucleotides with high-throughput sequencing	m6A m5C Ψ	● Determining the location of modification sites ● Single-nucleotide resolution techniques	● Potential false negatives ● Apparent mismatches from the expected sequence
High-throughput single-molecule sequencing	Directly measuring changes in base pairing like SMRT and Nanopore sequencing	m6A, m5C, hm5C Inosine Ψ	● Much longer sequencing-length ● Allow direct readout of modification sites ● Providing unbiased views of both the transcriptome and epitranscriptome ● Allowing direct quantitation of modification abundance	● Prone to noise and sequencing error ● Statistics problems ● Unmatured base-calling
*In silico* methods	High-throughput analysis of modified ribonucleotides	Various modifications	● Identifying modifications transcriptome-wide with single nucleotide resolution ● Retrospectively and can be readily applied to existing data and in meta analyses. ● Surveying multiple ● modification subtypes simultaneously	● Artifacts not be properly controlled Multiplesteps ● Limited to diploid and haploid organisms

m^6^A, *N*^6^-methyladenosine; m^5^C, 5-methylcytidine; m1A, *N*1-methyladenosine; Ψ, pseudouridine; hm5C, 5-hydroxymethylcytosine; SCARLET, site-specific cleavage and radioactive-labeling followed by ligation-assisted extraction and thin-layer chromatography; SMRT, single molecule real-time; snRNAs; small nuclear RNAs; snoRNAs, small nucleolarRNAs.

These high-throughput mapping methods have enabled the characterization of the epitranscriptome in diverse cellular environments. However, further technical improvements are needed to improve the resolution and sensitivity of these methods. Following the recent discovery of more writers, readers, and erasers of the epitranscriptome, as well as the detailed analysis of the known epitranscriptome, new research directions will emerge, which may lead to new treatment strategies. In the future, improved high-throughput technology should help improve spatial resolution, provide chemometric data, and possibly detect new mRNA modifications in the transcriptome.

### 5.2 Applied computational approaches for profiling RNA epitranscriptome

Although wet-lab approaches can obtain relatively accurate mRNA modification information, they are time-consuming, costly, and difficult to conduct. Given that RNA sequence numbers show explosive growth in the post-genomic era, wet-lab approaches are obviously not suitable for systematic and in-depth analysis of the relevant mechanisms and functions of RNA methylation modification. Therefore, many researchers have developed predictive and computational tools for identifying epigenetic modifications (Chen et al., 2020c; Guo et al., 2021), which have evolved rapidly in recent years (Rehman et al., 2021b,2022b; Liao et al., 2022). These tools are mainly based on machine learning (ML) or deep learning (DL) algorithms (Abbas et al., 2022). The information for most detection tools and databases has been described in recent reviews published by Chen et al. (2019b), El Allali et al. (2021) and Wang et al. (2022b). We have integrated the information from those studies with the most recent advances; this information is listed in Table 3. Furthermore, we categorized emerging tools into two types that identify single modification and multiple RNA methylation sites, respectively, based on the year that the tool developed (Table 3).

**TABLE 3:** Summary of tools for identifying RNA methylation sites.

Targets of RNA modification	Tools for identifying RNA methylation sites by years
*N*^6^-Methyladenosine (m6A)	● iRNA-Methyl (Chen et al., 2015a), m6Apred (Chen et al., 2015b) (2015) ● pRNAm-PC (Liu et al., 2016), RNA-MethylPred (Jia et al., 2016), AthMethPre (Xiang et al., 2016b), RNAMethPre (Xiang et al., 2016a), SRAMP (Zhou et al., 2016), TargetM6A (Li et al., 2016a), M6A-HPCS (Zhang et al., 2016), M6ATH (Chen et al., 2016a) (2016) ● MethyRNA (Chen et al., 2017a), RAM-ESVM (Chen et al., 2017b), RAM-NPPS (Xing et al., 2017) (2017) ● iMethyl-STTNC (Akbar and Hayat, 2018), M6APred-EL (Wei et al., 2018), RFAthM6A (Wang and Yan, 2018), BERMP (Huang et al., 2018), HMpre (Zhao et al., 2018), Zhang et al.’s (2018a) method, M6AMRFS (Qiang et al., 2018), DeepM6ASeq (Zhang and Hamada, 2018), iRNA (m6A)-PseDNC (Chen et al., 2018a), M6Apred-EL (Wei et al., 2018), m6ASNP (Jiang et al., 2018) (2018) ● Gene2vec (Zou et al., 2019), i*N*6-Methyl (5-step) (Nazari et al., 2019), WHISTLE (Chen et al., 2019a), DeepM6APred (Wei et al., 2019), FunDMDeep-m6A (Zhang et al., 2019), iRNA-Freq (Zhuang et al., 2019) (2019) ● LITHOPHONE (Liu et al., 2020e), WITMSG (Liu et al., 2020g), m6A-pred (Khan et al., 2020a), iRNA-m6A (Dao et al., 2020), im6A-TS-CNN (Liu et al., 2020c), iMethyl-deep (Mahmoudi et al., 2020), Pm6A-CNN (Alam et al., 2020), M6A-word2vec (Tahir et al., 2020), m6A Reader (Zhen et al., 2020) (2020) ● TS-m6A-DL (Abbas et al., 2021), M6A-GSMS (Zhang et al., 2021c), m6AGE (Wang et al., 2021), m6Aboost (Kortel et al., 2021), DNN-m6A (Zhang et al., 2021b), EDLm6APred (Zhang et al., 2021a), m6A-NeuralTool (Rehman et al., 2021a) (2021) ● DL-m6A (Rehman et al., 2022a), m6A-TSFinder (Song et al., 2022a) (2022)
5-methylcytosine (m5C)	● Feng et al.’s (2016) method, m5C-PseDNC (Feng et al., 2016) (2016) ● iRNAm5C-PseDNC (Qiu et al., 2017) (2017) ● pM5CS-Comp-mRMR (Sabooh et al., 2018), M5C-HPCR (Zhang et al., 2018b), PEA-m5C (Song et al., 2018), RNAm5Cfinder (Li et al., 2018a), M5C–HPCR (Zhang et al., 2018b) (2018) ● RNAm5CPred (Fang et al., 2019) (2019) ● iRNA-m5C_SVM (Dou et al., 2020b), m5CPred-SVM (Chen et al., 2020b), iRNAm5C_NB (Dou et al., 2020a) (2020) ● Staem5 (Chai et al., 2021) (2021) ● Li et al.’s (2022b) method (2022)
*N*^1^-methyladenosine (m1A)	● RAMPred (Chen et al., 2016b) (2016) ● ISGm1A (Liu et al., 2020f) (2020) ● m1ARegpred (Yao et al., 2022), m1A-Pred (Suleman and Khan, 2022) (2022)
*N*6, 2′-*O*-dimethyladenosine (m6Am)	● m6AmPred (Jiang et al., 2022), DLm6Am (Luo et al., 2022) (2022)
5-hydroxymethylcytosine (hm5C)	● iRNA5hmC (Liu et al., 2020i), iRNA5hmC-PS (Ahmed et al., 2020) (2020) ● iRhm5CNN (Ali et al., 2021), iR5hmcSC (Zhang and Shi, 2021) (2021) ● R5hmCFDV (Shi et al., 2022) (2022)
Pseudouridine (Ψ)	● tRNAmod (Panwar and Raghava, 2014) (2014) ● PPUS (Li et al., 2015b) (2015) ● iRNA-PseU (Chen et al., 2016c) (2016) ● PseUI (He et al., 2018) (2018) ● iPseU-CNN (Tahir et al., 2019), iPseU-NCP (Nguyen-Vo et al., 2019) (2019) ● RF-PseU (Lv et al., 2020b), iPseU–Layer (Mu et al., 2020), PIANO (Song et al., 2020b), EnsemPseU (Bi et al., 2020), PSI-MOUSE (Song et al., 2020a), MixedCNN-PseUI (Aziz and Hasan, 2020), MU-PseUDeep (Khan et al., 2020b) (2020) ● PA-PseU (Wang and Zhang, 2021), XG–PseU (Liu et al., 2020d), Aziz et al.’s model (Aziz et al., 2021), Porpoise (Li et al., 2021a), PseUdeep (Zhuang et al., 2021) (2021)
Multi-modification type prediction tools.	● HAMR (Ryvkin et al., 2013) (2013) ● iRNA-PseColl (Feng et al., 2017) (2017) ● iRNA-3typeA (Chen et al., 2018b) (2018) ● DeepMRMP (Sun et al., 2019) (2019) ● iMRM (Liu and Chen, 2020), DeepPromise (Chen et al., 2020c) (2020) ● iRNA-Mod-CNN (Tahir et al., 2021), MultiRM (Song et al., 2021b) (2021) ● EMDLP (Wang et al., 2022a), ZayyuNet (Abbas et al., 2022) (2022)

#### 5.2.1 Prediction tools for identifying m6A sites

Dao et al. (2020) established the iRNA-m6A tool based on the Support Vector Machine (SVM) with fivefold cross-validation test. It can accurate identify m6A sites with the data from high-throughput sequencing techniques in multiple human, mouse, and rat tissues. Liu et al. (2020c) further developed the im6A-TS-CNN tool and improved the results of iRNA-m6A using a convolutional neural network (CNN). Most recently, deep neural network (DNN)-based m6A site computation models, such as TS-m6A-DL, iMethyl-Deep, DNN-M6A, EDLm6APred, and DL-M6A, have been developed. These tools can achieve the identification of m6A methylation sites across species, and are specific to tissue and even RNA types (Mahmoudi et al., 2020; Abbas et al., 2021; Zhang et al., 2021a,b; Rehman et al., 2022a). Kortel et al. (2021) developed m6Aboost by combining ML with miCLIP (m6A individual-nucleotide resolution UV crosslinking and immunoprecipitation) to significantly improve the detection of m6A sites. miCLIP is a kind of antibody-based approach for m6A site mapping with single-nucleotide resolution. Zhang et al. (2021c) developed a novel predictor named M6A-GSMS based on the GBDT (Gradient Boosting Decision Tree) and stacking learning to identify m6A sites (Zhang et al., 2021c). At the same time, Wang et al. (2021) proposed the m6AGE predictor that combines sequence-derived features and graph embeddings for m6A site prediction. Recently, Rehman et al. (2021a) has made use of artificial intelligence to produce an effective model, the m6A-NeuralTool, which can be utilized for speedy and efficient identification of *N*^6^-methyladenosine sites (Rehman et al., 2021a). m6A TSHub is a comprehensive online platform established by Song et al. (2022a), which provides a web server constructed by multi-instance deep neural networks with gated attention for high-accuracy prediction of m6A methylation sites, named as m6A-TSFinder (Song et al., 2022a).

#### 5.2.2 Prediction tools for identifying m5C sites

Lv et al. (2020a) established a typical predictor named as iRNA-m5C based on the best features and random forest algorithm, to identify m5C sites in *Homo sapiens, Mus musculus, Saccharomyces cerevisiae, and Arabidopsis thaliana* (Lv et al., 2020a). Li et al. (2022b) developed a model to identify m5C based on a deep fusion approach with an improved residual network via 10-fold cross-validation and independent set testing, which shows a considerable improvement compared to previous tools (Li et al., 2022b).

#### 5.2.3 Prediction tools for identifying m1A sites

Yao et al. (2022) built the framework m1ARegpred (m1A regulators substrate prediction), m1ARegpred was achieved based on ML and the combination of sequence-derived and genome-derived features. Suleman and Khan (2022) developed an extreme gradient boost predictor named as m1A-Pred for the prediction of modified m1A sites.

#### 5.2.4 Prediction tools for identifying m6Am sites

Jiang et al. (2022) presented the m6AmPred, the first web server, for *in silico* identification of m6Am sites from the primary sequences of RNA. m6AmPred was built upon the XgbDart (eXtreme Gradient Boosting with Dart algorithm) and EIIP-PseEIIP encoding scheme. Luo et al. (2022) proposed an ensemble DL framework, named as DLm6Am, to identify m6Am sites. DLm6Am consists of three similar base classifiers, each of which contains a multi-head attention module, an embedding module with two parallel DL sub-modules, a CNN and a BiLSTM (Bidirectional long short-term memory), and a prediction module. Compared with the existing state-of-the-art m6Am prediction tool, m6AmPred and MultiRM show superior performance (Luo et al., 2022).

#### 5.2.5 Prediction tools for identifying hm5C sites

Zhang and Shi (2021) designed a novel and powerful model called iR5hmcSC for identifying hm5C. iR5hmcSC can achieve high-throughput identification of hm5C (Zhang and Shi, 2021). Shi et al. (2022) designed a model called R5hmCFDV. R5hmCFDV showed higher accuracy than iR5hmcSC does in the 10-fold cross-validation.

#### 5.2.6 Prediction tools for identifying Ψ sites

Zhuang et al. (2021) built PseUdeep, an RNA Pseudouridine Site Identification framework with DL Algorithm. PseUdeep outperformed the best traditional ML model available, which was evaluated through 10-fold cross-validation and two independent testing data sets (Zhuang et al., 2021).

#### 5.2.7 Prediction tools for multi-modification sites

Wang et al. (2022a) combined convolutional CNN and BiLSTM, and developed an ensemble multiscale DL predictor, EMDLP. It can identify RNA methylation sites by NLP (natural language processing) and DL way, and also take better advantage of the local and global information for site prediction. Abbas et al. (2022) proposed a unified DL model named as ZayyuNet, which can efficiently receive large inputs and achieve better performance based on its SpinalNet architecture that was inspired by the human somatosensory system.

### 5.3 Benchmark datasets related to RNA methylation

It is important to understand the methylation-related databases involved in developing these tools. We summarized the database information involved in methylation modification (Table 4) and sorted it into either single site or multiple site modification based on the year that the database was established in. Databases related to methylation modification are under development from comprehensive databases to more detailed and specialized ones. The following are the emerged databases of methylation modifications associated with some specific diseases and fields: (1) After m6A methylation-related genes based on The Cancer Genome Atlas (TCGA) were used to predict the prognosis of hepatocellular carcinoma (Group et al., 2020; Liu et al., 2020b; Li et al., 2021b), cancer-related methylation modification databases have begun to emerge, including, Lnc2Cancer 3.0 and OncoDB (Gao et al., 2021; Tang et al., 2022); (2) Osteoarthritis-omics and molecular biomarkers (OAOB), which are a group of database containing differential molecular biomarkers related to osteoarthritis (Li et al., 2022a); (3) Other than disease, Pan et al. (2022) established an integrative multi-omic database (iMOMdb) of Asian pregnant women providing the first blood-based multi-omic analysis of pregnant women in Asia. This database contains high-resolution genotypes, DNA methylation, and transcriptome profiles, and fills the knowledge gap of complex traits in populations of Asian ancestry; (4) Gao et al. (2022) developed AgingBank, an experimentally supported multiomics database of information related to aging in multiple species; (5) ProMetheusDB, a database generated by analyzing and sorting cell culture experiments data using ML tools from the protein perspective (Massignani et al., 2022); (6) compendium of protein lysine modifications (CPLM 4.0), a post-translational modification (PTMs) database (Zhang et al., 2022); (7) tRNA-related databases containing high-throughput tRNA sequencing data (Sajek et al., 2020); (8) RNAWRE and RM2 Target are two databases focusing on information of writers, readers, and erasers (Nie et al., 2020; Bao et al., 2022); and (9) SyStemCell, a multiple-levels experimental database for stem cell research (Yu et al., 2012). With the emergence of these specialized and multi-angle RNA methylation related databases, the traditional databases are also constantly updated and developed. Song et al. (2022a) established a comprehensive online platform, m6A-TSHub, to reveal context-specific m6A methylation and gene mutations that may regulate m6A epigenetic markers. Ma et al. (2022) proposed the M5C Atlas, a database for the comprehensive collection and annotation of RNA5 methylcytosine. Zhou et al. (2022) built ASMdb, a DNA modification database containing RNA sequencing data. Nevertheless, at present, specific databases for methylation modification such as m1A, m6Am, hm5C, and Ψ are still relatively lacking, which requires further improvement and development by researchers.

**TABLE 4:** RNA modification related database.

Targets of RNA methylation/modification	Database by years
*N*^6^-Methyladenosine (m6A)	● MeT-DB V2.0 (Liu et al., 2018), m6Avar (Zheng et al., 2018) (2018) ● CVm6A (Han et al., 2019) (2019) ● REPIC (Liu et al., 2020h) (2020) ● ConsRM (Song et al., 2021a), M6A2Target (Deng et al., 2021) (2021) ● m6A-TSHub (Song et al., 2022a) (2022)
5-methylcytosine (m5C)	● SyStemCell (Yu et al., 2012) (2012) ● m5C-Atlas (Ma et al., 2022) (2022)
*N*^1^-methyladenosine (m1A)	/
*N*6, 2′-*O*-dimethyladenosine (m6Am)	/
5-hydroxymethylcytosine (hm5C)	/
Pseudouridine (Ψ)	/
Database containing multiple types of RNA methylation/modification	● MODOMICS (Dunin-Horkawicz et al., 2006) ● TCGA (Wang et al., 2016) (2006) ● REACTOME (Croft et al., 2011), RNAMDB (Cantara et al., 2011) (2011) ● Gene-Expression Omnibus (GEO) (Barrett et al., 2013), DARNED (Kiran et al., 2013) (2013) ● RCAS (Uyar et al., 2017) (2017) ● RMBase V2 (Xuan et al., 2018), REDIdb 3.0 (Lo Giudice et al., 2018) (2018) ● RNAmod (Liu and Gregory, 2019) (2019) ● RNAWRE (Nie et al., 2020), T-psi-C (Sajek et al., 2020) (2020) ● m6A-Atlas (Tang et al., 2021), RMVar (Luo et al., 2021), Lnc2Cancer 3.0 (Gao et al., 2021) (2021) ● RMDisease V2.0 (Song et al., 2022b), AgingBank (Gao et al., 2022), CPLM 4.0 (Zhang et al., 2022), OncoDB (Tang et al., 2022), ASMdb (Zhou et al., 2022), iMOMdb (Pan et al., 2022), OAOB (Li et al., 2022a), ProMetheusDB (Massignani et al., 2022), RM2Target (Bao et al., 2022) (2022)

## 6 Conclusion and outlook

To provide biochemical researchers with the latest progress in RNA methylation, we reviewed the findings to date on the dynamic regulation and key roles of RNA methylation. We found that combining biochemical technology with high-throughput sequencing has made rapid progress in understanding the form and function of RNA modification, especially in mRNA and lncRNA. We mainly discussed m^6^A, m^5^C, m^1^A, pseudouridine, and 2’OMe. Other types of modifications that can be detected but are not clearly defined were not included. We also discussed the advantages and disadvantages of the detecting method based on high-throughput sequencing. It is worth mentioning that the databases related to RNA methylation modification, as well as the prediction and identification tools for RNA methylation sites developed with the help of these databases, have played an increasingly important role in RNA methylation modification research. We found that establishing a powerful method to investigate transcriptome modification is key to understanding post-transcriptional regulation. We emphasized that RNA methylation modification plays a key role in neural stem cells, synaptic functions, nervous system development, and brain development. Future research should focus on the role and mechanism of RNA methylation in neurodevelopmental disorders, which will greatly contribute to the prevention and treatment of developmental diseases.

## Author contributions

JZ and HL were responsible for gathering information and drawing the illustrations. YZ contributed to information interpretation, editing, and critical revision of the manuscript. WT, Y-QC, JD, S-YB, and Z-XW were responsible for revision of the manuscript. All authors read and approved the final manuscript.
